# Successful ceftazidime-avibactam treatment of MDR-KPC-positive *Klebsiella pneumoniae* infection in a patient with traumatic brain injury: A case report

**DOI:** 10.1097/MD.0000000000007664

**Published:** 2017-08-04

**Authors:** Agnese Gugliandolo, Carla Caio, Maria Lina Mezzatesta, Carmela Rifici, Placido Bramanti, Stefania Stefani, Emanuela Mazzon

**Affiliations:** a IRCCS Centro Neurolesi “Bonino-Pulejo”, Via Provinciale Palermo, Messina; b Section of Microbiology, Department of Biomedical and Biotechnological Sciences, University of Catania, Catania, Italy.

**Keywords:** ceftazidime-avibactam, chest trauma, Enterobacteriaceae, *Klebsiella pneumoniae*, traumatic brain injury

## Abstract

**Rationale:**

Carbapenem-resistant Enterobacteriaceae infections are a serious health care problem, because of the high mortality. Carbapenem resistance is mainly caused by carbapenemases production, including *Klebsiella pneumoniae* carbapenemase (KPC). Ceftazidime-avibactam is a new cephalosporin/β-lactamase inhibitor combination for the treatment of complicated urinary, intra-abdominal infections, and nosocomial pneumonia caused by gram negative, or other serious gram-negative infections.

**Patient concerns:**

We showed the case of a 27-year-old patient, hospitalized for traumatic brain injury and chest trauma, with KPC-producing *Klebsiella pneumoniae* infection.

**Diagnoses:**

Blood and bronchial aspirate culture analysis detected an infection caused by MDR *Klebsiella pneumoniae*, resistant to meropenem, ertapenem, piperacillin/tazobactam, amoxicillin/clavulanic acid, aztreonam, ceftazidime, cefotaxime, cefepime, amikacin, ciprofloxacin, trimethoprim/sulfamethoxazole, colistin while it showed an intermediate sensitivity to gentamicin and was sensitive to ceftazidime-avibactam. Molecular analyses revealed that the isolate belonged to the epidemic clone sequence type 258 (ST258) carrying *bla*_KPC-3_, *bla*_TEM-1_, and *bla*_SHV-11_genes.

**Interventions:**

After various combined antibiotic therapies without improvements, he was treated with ceftazidime-avibactam, on a compassionate-use basis.

**Outcomes:**

With ceftazidime-avibactam monotherapy clinical and microbiological clearance was obtained. A week after the end of the therapy microbiological analysis was repeated and a positive rectal swab for KPC-*Klebsiella pneumoniae* was found, becoming negative after 1 month. Moreover, the patient did not show any relapses for up to 18 weeks.

**Lessons:**

This case indicates that ceftazidime-avibactam monotherapy could be efficacious against KPC positive *Klebsiella pneumoniae* infections.

## 1 Introduction

Carbapenem-resistant Enterobacteriaceae (CRE) infections, especially *Klebsiella pneumoniae*, represent a major health care threat. The first reported carbapenem-resistant *K pneumoniae* was identified in the late 1990s in the United States and since that moment, CRE has disseminated globally, becoming endemic in some countries, including Italy. According to EARS-Net European surveillance system, the proportion of carbapenem-resistant *K pneumoniae* in our country was about 33.5% in 2015 (available at: http://atlas.ecdc.europa.eu/public/index.aspx).

The main mechanism of carbapenem resistance is the production of carbapenemases, mostly the *Klebsiella pneumoniae* carbapenemase (KPC). Most CRE infections occur in hospitalized patients, and the clinical outcome is usually poor. The mortality after CRE infection is high, approximately 40%,^[1]^ perhaps as a result of the limited treatment options.

Risk factors for CRE infections are other chronic diseases, the use of invasive medical devices, long-term hospitalizations, previous antibiotic administration, and journey to endemic regions. Dealing with antibiotic therapy, combination treatments are recommended,^[2]^ but data are insufficient to suggest the optimal regimen and the addition of colistin was reported to increase the cidal activity of these combinations.^[3]^ Furthermore, some therapies present limitations in their efficacy or toxicity.

Ceftazidime-avibactam (CAZ-AVI) is a new cephalosporin/β-lactamase inhibitor combination, approved with the trade name Zavicefta, for the treatment of complicated urinary tract, intra-abdominal infections, and nosocomial pneumonia due to gram-negative, or other serious gram-negative infections. In Italy, it is available only on a compassionate use basis and it is provided by Clinigen. Briefly, ceftazidime inhibits penicillin-binding proteins (PBP)_3_ of gram-negative bacteria, blocking cell wall synthesis, while avibactam inhibits Ambler Classes A, C, and some D β-lactamases, including the KPC and OXA-48 carbapenemases, but not metallo-β-lactamases.^[4]^ Avibactam alone did not possess antibacterial activity. CAZ-AVI showed a strong activity against multidrug-resistant (MDR) Enterobacteriaceae, CRE and *Pseudomonas aeruginosa*.^[4,5]^

## 2 Case report

A 27-year-old male was admitted to the intensive care unit of the IRCCS Neurolesi Center Bonino-Pulejo Messina (Italy) in November 2016, following a car accident, reporting traumatic brain injury. In particular, computed tomography (CT) revealed a double focal contusion hypodense area in the right cortical-subcortical, prefrontal, and fronto-temporal regions, associated with temporal-occipital extra axial effusion contralaterally. Moreover, it was observed a transverse fracture on the left petrous bone that reached the eardrum, in association with the subtotal occupation of mastoid cells. The patient showed also chest trauma, and CT analysis revealed the presence of a complete simple closed transverse fracture of the fifth left rib, without dislocation of bone fragments, with a focal hyperdense area above the diaphragm, in the medial segment of the left lower lobe. The patient's body weight was 128 kg and his medical history showed no previous infections and no comorbidities.

Since the day after hospitalization, the patient developed a low-grade fever (37.6°C), which increased gradually, reaching 39 to 40°C after a week. High fever (∼39–40°C) last for 5 days. After the temperature was stable at around 38°C. Blood tests showed an increase in white blood cell count (16.8 × 10^3^/μL) and in particular, neutrophils increased to 96.9% while lymphocytes decreased to 2.7%. Moreover, a decrease in red blood cells (3.61 × 10^6^/μL), hemoglobin (10 g/dL), hematocrit (30.9%), and platelet count (49 × 10^3^/μL) was observed. In addition, procalcitonin concentration was 63.1 ng/mL, but the range is 0 to 0.1 ng/mL.

The fever was associated with a pulmonary infection, confirmed by CT scan, that revealed subpleural consolidations in the dorsal segment of the upper right lobe and in the apical segment of the lower right lobe. Moreover, bronchial aspirate, venous central catheter tip, and blood culture analysis were positive for MDR *K pneumoniae* isolates according to the bioMérieux Vitek-2 automated system, resistant to all antibiotics.

Waiting for a deep characterization of the *K pneumoniae* isolates and for the confirmation of the resistance profile, the following therapies were administered to the patient: ampicillin/sulbactam (i.v. 2 g/1 g every 8 hours), levofloxacin (i.v. 500 mg/d), vancomycin (i.v. 500 mg every 6 hours), colistin (90,00,000 U/d), meropenem (i.v. 1 g every 8 hours), rifampicin (i.v 600 mg/d), linezolid (i.v. 600 mg every 12 hours), and tigecycline (i.v. 100 mg every 12 hours), but without improvements. The combinations of antibiotics administered to the patient are reported in Table 1. The therapy with tigecycline was interrupted because the patient developed pancreatitis as an adverse event, with increased levels of amylase and lipase. At the same time samples were sent to the Laboratory of Molecular Microbiology and Antibiotic Resistance of the University of Catania for the confirmation of the resistance profile of the isolate. MIC determinations of the following antibiotics were performed by gradient test (Liofilchem, Roseto degli Abruzzi, Italy): meropenem, ertapenem, piperacillin/tazobactam, amoxicillin/clavulanic acid, aztreonam, ceftazidime, cefotaxime, cefepime, amikacin, gentamicin, ciprofloxacin, trimethoprim/sulfamethoxazole, colistin, tigecycline, and CAZ-AVI. Susceptibility and resistance categories were assigned according to the European Committee on Antimicrobial Susceptibility Testing (EUCAST) breakpoints (available at: http://aurosan.de/wp-content/uploads/2015/05/v_6.0_Breakpoint_table.pdf.). *Escherichia coli* ATCC 25922 was used as the quality control strain. Phenotypic screening for carbapenemases or overexpression of AmpC in combination with porin loss in *K pneumoniae* strains was performed by a commercial synergy test (RoscoDiagnostica, Taastrup, Denmark). Oropharyngeal and rectal swabs, and blood culture analysis were positive for *K pneumoniae.* The isolate was positive for KPC production and showed a MDR profile; in fact, it was resistant to all antibiotics except CAZ-AVI with a MIC value of 1 mg/L, and it showed an intermediate sensitivity to gentamicin with MIC value of 4 mg/L. CAZ-AVI was not available in Italy, and then the procedures for the request for compassionate use were made, with the permission of the requesting hospital. In the meantime, the patient was treated with gentamicin (1 mg/kg every 8 hours), obtaining a decrease of the temperature. After the request for compassionate use, CAZ-AVI was kindly provided by Clinigen. The supply of CAZ-AVI has been approved by the ethical committee (approval n. 13/16). Ceftazidime 2 g/ avibactam 0.5 g was administered every 8 hours for 14 days.

**Table 1:**
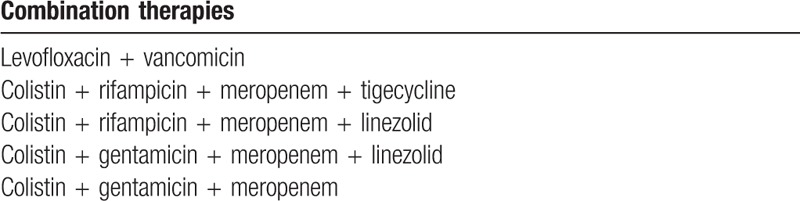
Combination therapies administered to the patient before ceftazidime-avibactam treatment.

Since 2 days after CAZ-AVI administration fever disappeared. Moreover, phlogosis indices (reactive C protein: 0.5; procalcitonin: 0.05 ng/mL) and white blood cells (∼7.3×10^3^/μL) decreased.

The patient kidney function was always monitored, given that this is the route of CAZ-AVI elimination, and so it is necessary a dose adjustment in the case of renal impairment, given that half-life and clearance are expanded in subjects with renal problems.^[4]^ However, in our patient no alterations were found (creatinine: 0.6 mg/mL; creatinine clearance: 131 mL/min; uricemia: 4.5 mg/dL; azotemia: 21 mg/dL). No adverse events were reported during CAZ-AVI treatment.

In the meantime, the *K pneumoniae* isolate was subjected to molecular analysis to better characterize the MDR profile. Identification of genes encoding carbapenemases (*bla*_VIM_*, bla*_*I*MP_*, bla*_NDM_*, bla*_KPC_, and *bla*_OXA-48_ genes), extended-spectrum ß-lactamases (ESBLs) (such as *bla*_SHV_*, bla*_TEM_*, bla*_CTX-M_), and plasmid-mediated AmpC beta-lactamases was performed by PCR and sequencing as previously described.^[6–9]^ Sequences were analyzed using the BioEdit software and BLAST tool (http://www.ncbi.nlm.nih. gov/BLAST). *K pneumoniae* isolates carried the *bla*_*KPC-3*_, *bla*_TEM-1_genes, and were positive for *bla*_SHV-11_gene. Multilocus sequence typing (MLST) was carried out by PCR and sequencing according to Diancourt et al,^[10]^ and sequence types were analyzed using the Institute Pasteur database (http://bigsdb.pasteur.fr/perl/bigsdb/bigsdb.pl?db = pubmlst_klebsiella_seqdef_public). Analyses revealed that the isolate belonged to the epidemic clone sequence type 258 (ST258). To find the localization of KPC-3, PCR assays with specific primers and sequencing were performed as previously published.^[11,12]^ KPC-3 was found as part of the 10 kb Tn3-like element Tn4401 in pKpQIL plasmid, as classically in ST258.

**Figure 1:**
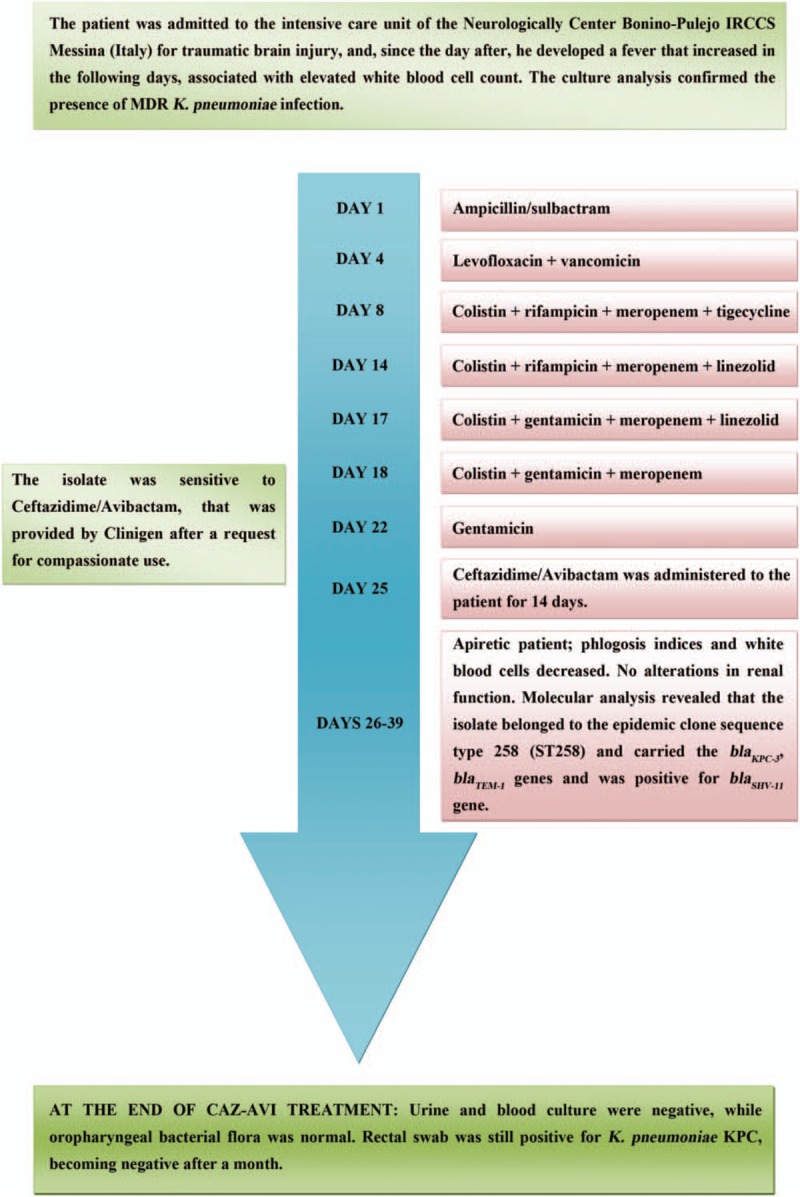
Timeline indicating the diagnosis, the different therapies administered to the patient and the final result after CAZ-AVI treatment. CAZ-AVI = ceftazidime-avibactam, KPC = *Klebsiella pneumonia* carbapenemase, MDR = multidrug-resistant.

A week after the end of the therapy, urine, oropharyngeal and rectal swabs, and blood culture analysis were repeated. Urine and blood culture were negative, while oropharyngeal bacterial flora was normal. Only rectal swab was still positive for *K pneumoniae* KPC. A month after, a check was carried out for the possible presence of KPC positive *K pneumoniae*, and rectal and oropharyngeal swabs, and blood culture analysis were repeated, but the patient was not even colonized. Indeed, also rectal swab became negative. Also the following month blood culture, rectal and oropharyngeal swabs were negative for *K pneumoniae*. Moreover, each month the patient repeated rectal and oropharyngeal swabs analysis. We found that oropharyngeal bacterial flora was normal, while rectal swab was negative. Then, no recurrence of infection was reported within 18 weeks, as confirmed by microbiological analysis. Moreover, blood test showed that white blood cell count and phlogosis indices were in the normal range. A timeline indicating the diagnosis, the therapies administered to the patient and the final result is shown in Fig. 1.

## 3 Discussion

KPCs-producing *K pneumoniae* infections are associated with high morbidity and mortality rates,^[13]^ and are widely disseminated in Italy. Indeed, an Italian analysis of 131 *K pneumoniae* isolates from hospitalized patients revealed that about 30% of them were resistant to at least 1 carbapenem antibiotic, suggesting the spreading of Carbapenem-resistant *K pneumoniae* isolates. In particular, all these isolates produced carbapenemases and almost all the isolates carried the bla_KPC_ gene.^[14]^ Especially, *K pneumoniae* ST258 is associated with KPC production and resistance to different antibiotics.^[15]^

Therapies against carbapenemase-producing *K pneumoniae* are limited and based mainly on colistin, tigecycline, and aminoglycosides, including gentamicin.^[16]^ However, different reports indicate that combination therapies are more effective compared with monotherapy. Tumbarello et al^[17]^ showed that combination therapy was associated with a lower mortality rate. In particular, in a cohort of 125 patients with KPC-producing *K pneumoniae* bloodstream infections, 22 of them were treated with colistin, 19 with tigecycline, and 5 were administered with gentamicin, but the majority of the patients received 2 or more drugs that showed in vitro activity against the isolate. The most used combination therapy was colistin with tigecycline, with or without meropenem. Notably, the administration of these 3 antibiotics was the most common in the survivor group.^[17]^ The same authors confirmed the superiority of combination therapy in a larger cohort of patients with bloodstream, lower respiratory tract, intra-abdominal, urinary tract, and other infections caused by KPC-producing *K pneumoniae*.^[18]^ Similar results were reported by Qureshi et al^[19]^ indicating that combination therapies were associated with a lower mortality (13.3%) compared with monotherapy (57.8%) in a cohort of 41 patients with bacteremia caused by KPC-producing *K pneumoniae*. The most used combinations were colistin-polymyxin B or tigecycline with a carbapenem. Instead, the monotherapy consisted of colistin-polymyxin B, tigecycline, or carbapenem (imipenem or meropenem). In particular, patients receiving colistin-polymyxin B or tigecycline alone showed a higher mortality, although the in vitro susceptibility.^[19]^ In addition, in a study with 162 patients with *K pneumoniae* bloodstream infections (14 of them were infected with carbapenems resistant VIM-1-producing *K pneumoniae*) the mortality rate was lower in patients receiving combination therapy with 2 active drugs (one was a carbapenem and the other colistin or an aminoglycoside) compared with patients treated only with 1 active drug (a carbapenem, colistin, or an aminoglycoside).^[20]^ According to a report, combination therapies were more effective than monotherapy and in particular those containing carbapenem were associated with a lower mortality.^[21]^ However, it is not easy to define the most adapt treatment and some therapies may show limits in their efficacy or adverse effects.

CAZ-AVI is approved for the treatment of complicated urinary tract and intra-abdominal infections, hospital-acquired pneumonia, and gram-negative infections. In this combination, avibactam, being a β-lactamase inhibitor, restores ceftazidime activity against ESBLs and CRE Enterobacteriaceae. In vitro and in vivo models indicate the efficacy of CAZ-AVI for the treatment of complicated infections caused by CRE and MDR and ESBL-positive *K pneumoniae* isolates.^[5,22–24]^

In this report, we showed the case of a critically ill patient with MDR-*K pneumoniae* infection, resistant to all antibiotics except CAZ-AVI (MIC: 1 mg/L) and with intermediate sensitivity to gentamicin (MIC: 4 mg/L), treated with CAZ-AVI. Different combination therapies were administered to the patient, but with no improvements. Interestingly, the patient was treated with CAZ-AVI monotherapy, and since the second day of treatment, his conditions significantly improved. Indeed, white blood cell count and inflammation indices decreased and the patient was apiretic. This suggests the great potential of CAZ-AVI monotherapy in the treatment of *K pneumoniae*/KPC positive infections. At the end of the therapy only rectal swab was positive, but after a month it became negative, indicating that the patient was not even colonized. This suggests a great effect of CAZ-AVI on the eventual colonization. Notably, no recurrence of the infection was observed within 18 weeks, confirmed by the microbiological analysis. Indeed, blood cultures were repeated for 2 months after CAZ-AVI treatment and they were negative, while rectal and oropharyngeal swabs were repeated each month after CAZ-AVI treatment. Rectal swab was negative, while oropharyngeal bacterial flora was normal. In addition, white blood cell count and phlogosis indices were in the normal range. In addition, no nephrotoxicity was observed. However, kidney function must be monitored during CAZ-AVI treatment given that it is necessary a dose adjustment in patients with renal problems.^[4]^ Only a case report investigated the pharmacokinetic data from 2 patients with KPC-producing *K pneumoniae* bloodstream infections with renal impairment, where 1 was obese, and in both patients larger volumes of distribution than those reported were observed, suggesting the need to define the optimal dose for renal impaired and obese patients.^[25]^

In the last years, reports demonstrated that CAZ-AVI treatment is efficacious in combination with other antibiotics to treat CRE and in particular *K pneumoniae* infections.

A combination of intravenous CAZ-AVI and ertapenem was efficacious in a 64-year-old patient with pandrug-resistant carbapenem-resistant *K pneumoniae* bacteremia.^[26]^ In particular, the patient was hospitalized for an intestinal transplant. After the transplant, the patient developed a complicated intra-abdominal infection caused by carbapenem-resistant *K pneumoniae*. Also the bronchoalveolar lavage fluid, blood, urine, and central venous catheter tip cultures were positive for carbapenem-resistant *K pneumoniae*. Different antimicrobial therapies failed in the treatment of the infection. However, after the treatment with CAZ-AVI and ertapenem the patient showed sterilization of blood cultures after 24 hours. The patient received the treatment for 2 weeks and no adverse events were observed during the therapy.^[26]^ A case report documented CAZ-AVI treatment administered as extended infusion in combination with colistin and tigecycline in a kidney and pancreas transplant 47-year-old patient with bacteremia due to KPC-3 *K pneumoniae*.^[27]^ Blood cultures remained positive for 48 hours, but clearance was reported after femoral line exchange. However, the patient's conditions were really critical, because of the presence of bacteremia and abdominal abscess, and finally the patient died because of cardiopulmonary arrest.^[27]^

In addition, CAZ-AVI monotherapy was able to cure 3 patients with CRE infections, 2 of them positive for *K pneumoniae,* and 1 for *Enterobacter aerogenes*, obtaining the eradication of the pathogens. This result is interesting considering that 2 of the patients had complicated infections. In particular, 1 patient was in septic shock, and the other had an endocarditis.^[28]^ This case is in line with ours, indicating the efficacy of CAZ-AVI monotherapy in the treatment of complicated infections.

Moreover, it is important to notice that the patient in our case report is a young adult, and this may have positively influenced the success of the treatment. Indeed, in general, older patients may present a greater risk of infections or of unsuccessful treatments, associated with an increased risk of complications or of dying, and more adverse effects as a consequence of therapies. In particular, advanced age was shown to be an independent predictor of negative outcome in patients with *K pneumoniae* bloodstream infections,^[20]^ and age>50 years was associated with increased mortality in patients with ventilator-associated pneumonia caused by CRE.^[29]^ However, we can suppose that CAZ-AVI treatment could be effective also in older patients. Indeed, in the other case reports showing the successful treatment with CAZ-AVI, in combination with ertapenem or as monotherapy, patients were 64 or more than 70 years old, respectively. ^[26,28]^

Some patients can show a recurrence of infection. A report evaluating the recurrence of bacteremia caused by ESBL *K pneumoniae* indicates that in the majority of cases the recurrent infection was caused by a genetically similar isolate and the mean of the time between the 2 episodes was about 30 days.^[30]^ In a cohort of 44 patients with CRE infections, 6 patients with carbapenem-resistant *K pneumoniae* infections were treated with CAZ-AVI, but only in 2 of them as monotherapy. Five patients obtained clinical cure, but 2 of them, receiving a combination therapy, relapsed with the same strain within 3 weeks after CAZ-AVI treatment and died because of sepsis. However, it is important to notice that most of the patients were septic and presented comorbidities, such as renal impairment, that may have influenced the negative clinical outcomes.^[31]^ On the contrary, we show the case of a patient with pneumonia successfully treated with CAZ-AVI monotherapy with no relapse of infection up to 18 weeks, confirmed by the microbiological analysis, indicating the efficacy of CAZ-AVI treatment in our case.

In conclusion, this case, demonstrating the successful treatment of *K pneumoniae* infection in a critically ill patient, indicates the efficacy of CAZ-AVI monotherapy for MDR-KPC-positive *K pneumoniae* infections. Notably, we reported no relapse within 18 weeks, suggesting the potential of CAZ-AVI treatment in the eradication of the pathogen. Therefore, CAZ-AVI increases the antimicrobial therapeutic approaches against MDR gram-negative pathogens, in particular *K pneumoniae,* and its expanded activity against ESBLs and CREs is notable.
